# *Morus alba* L. and *Morus nigra* L. Leaves as a Promising Food Source of Phenolic Compounds with Antioxidant Activity

**DOI:** 10.1007/s11130-021-00922-7

**Published:** 2021-09-27

**Authors:** Milena Polumackanycz, Marek Wesolowski, Agnieszka Viapiana

**Affiliations:** Department of Analytical Chemistry, Medical University of Gdansk, Al. Gen. Hallera 107, 80-416 Gdansk, Poland

**Keywords:** Mulberry leaves, Phenolic composition, Antioxidant activity, Correlation analysis

## Abstract

Bioactivity of mulberry has been widely described, but mostly related to its fruits, while studies with leaves are scarce. Herein, in this study leaves of two mulberry species (*Morus alba* L. and *Morus nigra* L.) were characterized, compared, and evaluated in terms of their phenolic composition and antioxidant activity. Aiming to valorize mulberry leaves extracts which can be included in modern diet four different extracts (infusions, decoctions, tinctures and hydromethanolic) were examined. The chemical characterization was done by quantifying total phenolics (TPC), flavonoids (TFC) and phenolic acids (TPAC) content, L( +)ascorbic acid (ASA) and individual phenolic compounds in the extracts by HPLC technique. Moreover, DPPH and FRAP assays were used to assess the antioxidant activity of white and black mulberry leaves. Findings of these studies revealed that black mulberry leaves were richer in TFC and TPAC, while white mulberry leaves contained higher levels of individual phenolic compounds in water extracts. Gallic acid was found in the highest concentrations in two mulberry leave extracts. Correlation analysis showed strong relationships between antioxidant activity and TPC and TFC. This fact suggests the crucial role of phenolic compounds as antioxidant agents in white and black mulberry leaves. The results obtained in this study demonstrate that leaves of both white and black mulberry can be used as a valuable source of phenolic compounds with bioactive potential, which can be applied in the food sector, as foods and as promising source of natural ingredients.

**Supplementary Information:**

The online version contains supplementary material available at 10.1007/s11130-021-00922-7.

## Introduction

The genus *Morus* (Moraceae) consists of approximately 19 members, which are mainly distributed in the north temperate zone. The most commonly species are white mulberry (*Morus alba* L.), black mulberry (*Morus nigra* L.), and red mulberry (*Morus rubra* L.) [1]. Among these species, *Morus alba* is the dominant one. Mulberry is widely cultivated in Turkey, South Europe, Central and Southwest Asia [2]. Various morphological parts of this plant (leaves, fruits, roots, stems) have been used for different purposes. Herein, the vast majority of the content focuses on mulberry fruits and leaves, which have got medicinal properties and are often consumed as part of a typical diet.

Leaves of mulberry have been used as tea and powder juice [3]. In some Asian countries they are used as nourishment. In Korea mulberry leaves are one of the ice-cream ingredients, while in India they are a good nutritious, non-toxic and low cost food compound for paratha, the traditional meal item of breakfast and dinner of the Indian diet [4]. Moreover, in Japan and Korea patients with diabetes consume mulberry leaves as an anti-hyperglycemic supplement [5]. Mulberry leaves are effective against high blood pressure and hangover from alcohol and they are prevented throat infections, irritations and inflammations. Over the past decades the consumption of mulberry tea has increased because of its hypoglycemic, antidepressant, antioxidant and hepatoprotective effects [6]. Currently, mulberry leaves were authorized as an excellent food resource with high content of protein, carbohydrate, vitamins, microelements and dietary fiber. Besides some reports indicated that they are rich in phenolic acids, flavonoids, alkaloids, and γ-aminobutyric acid (GABA) [7, 8]. These bioactive compounds have possess anti-HIV, antioxidative, hypotensive, cytotoxic [9], hypoglycemic [10, 11], hepatoprotective [10, 11], neuroprotective [12] and anti-inflammatory [13] properties. Moreover, they have also been applied in antibacterial [6, 14] and anti-obesity [15] treatments.

Subsequently, as a source of pharmacologically active compounds, particularly in the search for drugs for many diseases, plants continue to be used with phytotherapeutic activities and other industrial applications. One important activity for some plants is the free radical-scavenging power, which is also crucial for the food industry. Based on the above considerations, in this study leaves of two mulberry species (*Morus alba* L. and *Morus nigra* L.) were characterized, compared and evaluated, in terms of their phenolic composition and antioxidant activity, aiming to valorize mulberry leaves extracts which can be included in modern diet.

## Materials and Methods

### Chemicals and Plant Materials

Wild samples of *Morus alba* L. and *Morus nigra* L. leaves were collected during August 2018 in Bari, Italy. The collected plant materials were authenticated by Professor Pinarosa Avato from Dipartimento di Farmacia-Scienze del Farmaco, Universita degli Studi di Bari, Italy. The leaves were pulverized in a water-cooled Knifetec 1095 grinder (Foss Tecator, Höganäs, Sweden) and the homogenized samples were stored in a fresh and dry place, away from any light source until further analysis.

### Preparation of Mulberry White and Black Extracts

Hydromethanolic extracts were prepared by sonication of 1 g sample with 4 mL of methanol–water mixture (80:20, v/v) for 10 min at 20 °C using an ultrasonic bath (Emag, Salach, Germany). The suspension was centrifuged in an EBA-20S centrifuge (Hettich, Tuttlingen, Germany) for 5 min at 8,000 rpm and the supernatant transferred into a 20 mL volumetric flask. This procedure was repeated twice, the extracts obtained were combined and diluted up to 20 mL with a mixture of methanol–water (80:20, v/v). Infusions were obtained by adding 200 mL of boiling water (100 °C) to 1 g sample of mulberry. The mixture was then left to stand at room temperature for 10 min. Decoctions were performed by adding 200 mL of distilled water to the sample (1 g), boiled for 5 min, left to stand for 5 min. To prepare the tinctures, 3 g samples were macerated with 50 mL of an ethanol–water (70:30, v/v) mixture, left for 7 days at room temperature, and occasionally shaken to maximize extraction. The infusions, decoctions and tinctures were filtered through a Whatman paper (Macherey–Nagel, Duren, Germany), transferred into graduated flask and diluted with a solvent up to 50 mL. Prior to HPLC analysis, the extracts were also filtered through a 0.22-pm nylon membrane filter (Witko, Lodz, Poland).

### Determination of Phenolics and L( +)-Ascorbic Acid Content

Total phenolics content (TPC) of the mulberries extracts was determined using the Folin-Ciocalteu method described by Singleton and Rossi [16] with some modifications. The TPC of the extracts was expressed in mg gallic acid equivalent *per* g dried weight of sample (mg GAE/g DW). Total flavonoids content (TFC) of the mulberries extracts was determined by the method described in the European Pharmacopoeia [17] with some modifications. The TFC was expressed as μg of quercetin equivalent *per* g dried weight of sample (μg QE/g DW). The procedure described in the Polish Pharmacopoeia VI [18] was used for total phenolic acids content (TPAC) determination with Arnov’s reagent. The results were expressed in μg of caffeic acid equivalent *per* g dried weight of sample (μg CAE/g DW). The Abdelmageed et al. method [19] was used for L( +)-ascorbic acid content (ASA) determination. The content of L( +)-ascorbic acid in the extract was expressed as mg of ascorbic acid per g dried weight of sample (mg ASA/g DW). Detailed description of this method is provided in the Supplementary material.

The chromatographic separation, identification and quantification of 10 phenolic constituents: gallic acid (GA), caffeic acids (CA), *p*-coumaric acid (*p*CA), ferulic acid (FA), sinapic acid (SYN), rosmarinic acid (RA), chlorogenic acid (CGA), rutin (RUT), myricetin (MYR) and naringenin (NAR) were performed using a HPLC–UV/Vis system (LaChrom, Merck, Darmstadt, Germany) [20]. The analysis was carried out at 30 °C, with acetonitrile-0.5% acetic acid solution (solvent A) and water-0.5% acetic acid solution (solvent B) as mobile phase. A gradient program was choosen as follow: 0–10 min, linear 5–15% A; 10–15 min, linear 15–20% A; 15–20 min, linear 20–30% A; 20–25 min, linear 30–63% A; 25–30 min, isocratic 63% A; 30–35 min, linear 63–5% A. The flow rate of mobile phase was 1.0 mL/min, and the runs were monitored at 280 nm for GA, RA, SYN and NAR, at 320 nm for CA, CGA, *p*CA and FA, and at 360 nm for RUT and MYR. Analytes were identified comparing their retention times with the standard compounds. Additionally, a selected sample was spiked with the standard compounds and analyzed again.

The validation parameters for HPLC procedure are listed in Table 1. Detailed inspection of the data shows that precision of the HPLC procedure was acceptable, CV values ranging between 0.20 and 3.76%, and 0.26 and 6.02%, for intra- and inter-day variations, respectively. For the stability test, retention CV was lower than 1.7% for peak area and 0.6% for retention time. Apart from this, peak areas and retention times of phenolic compounds were found to be sufficiently stable over 48 h.

**Table 1:** Validation parameters of the calibration curves for quantified phenolic compounds

	Regression equation	Linearity (μg/mL)	R^2^	LOD (μg/mL)	LOQ (μg/mL)	Recovery (%)
GA	y = 52045x—482,462	23.1–116.7	0.996	3.05	10.06	94.53
CGA	y = 3082x + 47,036	22.5–105.1	0.990	2.51	6.94	92.67
RA	y = 3007x + 48,565	20.4–120.4	0.991	2.76	7.86	97.86
CA	y = 4733x + 108,103	25.2–127.7	0.989	3.70	10.05	102.64
SA	y = 6213x + 114,473	23.1–113.5	0.992	6.43	13.11	95.34
*p*CA	y = 10400x + 89,291	21.8–105.5	0.998	4.32	13.06	97.11
FA	y = 14076x—84,688	23.4–117.6	0.999	5.21	10.98	94.67
CIN	y = 5239x—28,982	22.5–102.6	0.998	3.32	9.67	103.53
RUT	y = 5223x + 118,806	23.2–115.4	0.992	3.65	9.54	110.46
MYR	y = 17076x—71,212	28.8–112.1	0.998	4.65	13.77	96.96
NAR	y = 10128x—57,109	25.5–128.4	0.990	3.57	10.86	97.38

x refers to the concentration of compound (μg/mL)

y is the peak area

### DPPH Scavenging Activity Assay and FRAP Assay

In this study DPPH and FRAP tests were used to validate the antioxidant activity of mulberry leaves samples. Both tests are recommended as rapid, simple, cheap and reproducible tools for measuring the antioxidant activity of the plants. The DPPH radical scavenging activity was assessed according to a modified method of Tuberoso et al. [21], whereas ferric reducing/antioxidant power (FRAP) assay was performed using the method proposed by Benzie and Strain [22]. The results were expressed as mg Trolox equivalent *per* 100 g dry weight of sample (mg TE/100 g DW) for DPPH test, and mmol ferrous ion equivalent *per* g dry weight of sample (mmol Fe^2+^/g DW) for FRAP test. Detailed description of DPPH and FRAP methods is presented in the Supplementary material.

### Statistical Analysis

The analyses were carried out at least in triplicate and the results were expressed as arithmetical mean ± standard deviation (SD). The data were analyzed using one-way analysis of variance (ANOVA) test, followed by Tukey HSD test. The relationship between phenolic compounds of different mulberry extracts and antioxidant activity was analyzed by a Pearson correlation analysis. Statistical data analysis was performed using Statistica 10 software (StatSoft Inc., Tulsa, USA) using parametric test with the level of significance of *p* < 0.05.

## Results and Discussion

### Phenolic Composition

Phenolic compounds are the most frequently examined phytochemicals in plants because of their health benefits. A daily diet enriched in these compounds is important to promote wellbeing. Moreover, phenolic compounds are currently considered an indispensable component in a variety of cosmetic, nutraceutical, and pharmaceutical applications owing to their anti-carcinogenic, antioxidative, anti-inflammatory, and anti-mutagenic activity [23].

Quantification of the TPC, TFC, TPAC and ASA in white and black mulberry leaves (Table 2, Fig. 1S) revealed that extracts prepared from black mulberry were richer in TFC and TPAC compared to white mulberry extracts, while TPAC of hydroalcoholic extracts of black mulberry were several times higher than those in extracts of white mulberry. This is reflected by the statistically significant differences (*p* < 0.05) between water extracts of both mulberry species. However, infusions and decoctions prepared from white and black mulberry were richer in phenolic compounds than their tinctures and hydromethanolic extracts. In the case of ASA content no significant differences were found (*p* < 0.05) between white and black mulberry leaves, excluding their decoctions. To the best of authors knowledge, this is the first report of the TPAC and ASA in mulberry water and hydroalcoholic extracts.

**Table 2:** The content of total phenolics (TPC), flavonoids (TFC), phenolic acids (TPAC), and DPPH and FRAP values of white and black mulberry leaves. Results as presented as arithmetic mean ± standard deviation (SD)

	Infusions	Decoctions	Tinctures	Hydromethanolic extracts
*Morus alba* L				
TPC	2.14 ± 1.15^c^	5.36 ± 0.60^e^	0.26 ± 0.03^a^	0.31 ± 0.03^a^
TFC	1.37 ± 0.07^b^	4.21 ± 0.10^d^	0.16 ± 0.01^a^	0.27 ± 0.05^a^
TPAC	1.13 ± 0.38^b^	1.06 ± 0.16^b^	0.25 ± 0.01^a^	0.13 ± 0.01^a^
ASA	0.54 ± 0.10^a^	0.48 ± 0.04^a^	0.17 ± 0.08^a^	0.55 ± 0.02^a^
DPPH	1.46 ± 0.03^c^	3.96 ± 0.78^b^	0.09 ± 0.01^a^	0.19 ± 0.04^a^
FRAP	15.79 ± 0.23^b^	16.68 ± 1.59^b^	1.06 ± 0.06^a^	1.34 ± 0.87^a^
*Morus nigra* L				
TPC	3.26 ± 0.75^e^	5.19 ± 1.068^f^	0.17 ± 0.04^a^	0.54 ± 0.02^ab^
TFC	21.10 ± 0.60^d^	26.21 ± 0.94^d^	0.98 ± 0.03^bc^	0.43 ± 0.01^ab^
TPAC	14.92 ± 0.18^c^	11.75 ± 0.22^bc^	7.74 ± 0.05^ g^	2.53 ± 0.06^d^
ASA	0.58 ± 0.24^ab^	1.29 ± 0.18^c^	0.13 ± 0.06^a^	0.26 ± 0.05^a^
DPPH	4.31 ± 0.87^b^	2.29 ± 0.21^d^	0.16 ± 0.13^a^	0.09 ± 0.01^a^
FRAP	6.42 ± 0.65^c^	10.75 ± 1.06^d^	1.10 ± 0.53^a^	1.18 ± 0.07^a^

TPC is expressed as mg GAE/g DW; TFC is expressed as μg QE/g DW; TPAC is expressed as μg CAE/g DW; ASA is expressed as mg ASA/g DW; DPPH is expressed as mg TE/g DW and FRAP is expressed as mmol Fe^2+^/g DW

Arithmetic means followed by the same letter within a row indicate no significant difference (*p* < 0.05) in Tukey test

The findings of this work were in agreement with a report on mulberry leaves from Pakistan [24], which also indicated higher TPC values for black mulberry than for white mulberry. However, Sanchez-Salcedo et al. [7] found TPC in the same level, over the dozen mg GAE/g DW, in white and black mulberry leaves from different clones grown in Spain. Also Bazylak et al. [25] found TPC and TFC on the levels over 600 mg CAE/100 g DW and 302.70 mg CAE/100 g DW, respectively, in water extracts of white mulberry leaves originating from Poland, China and Bulgaria. These data are similar to those obtained in this study. Moreover, Radojkovic ´ et al. [26] reported higher values of TPC in mulberry leaves, from 66.76 to 115.23 mg GAE/g DW for white and black mulberry, respectively. Kim et al. [27] revealed TFC in the range from 28.2 to 55.4 mg GAE/g DW in methanol extracts of white mulberry leaves, while Memon et al. [28] obtained TPC on the level of 8.33 mmol/100 g DW in white mulberry leaves. These values are higher than those obtained in this study. An explanation behind the variability among the phenolic contents in mulberry leaves could be found due to the different extraction procedures and analytical methods used in each work. In addition, it has been suggested that phenolic compounds in leaves vary according to conditions such as drought, temperature changes, pollution, UV light, and pathogen attacks, among others [29].

The contents of individual phenolic compounds in different extracts of white and black mulberry leaves are compiled in Table 3 and HPLC chromatograms are shown in Fig. 2S. Eleven phenolic compounds were identified and quantified: seven phenolic acids (GA, CGA, RA, CA, *p*CA, FA, SYN) and three flavonoids (RUT, MYR, NAR). The significant differences were found between the content of phenolic compounds in extracts of white and black mulberry. Overall, higher amounts of phenolic acids and flavonoids were found in white mulberry than black mulberry extracts. However, in both species of mulberry water extracts were richer in phenolic compounds than their alcoholic extracts. GA was the most abundant phenolic constituent in all extracts. RA and RUT were found on the higher levels in white mulberry extracts, while CA was predominated in black mulberry extracts. *p*CA were found in the lowest amounts in the most of mulberry extracts, while CGA was not detected in decoctions and hydromathanolic extracts of white mulberry leaves.

**Table 3:** The content of phenolic compounds in different extracts of white and black mulberry leaves

	Infusions	Decoctions	Tinctures	Hydromethanolic extracts
*Morus alba* L	mg/g	mg/g	μg/g	μg/g
gallic acid	4.34 ± 0.56^b^	2.00 ± 0.12^a^	179.35 ± 2.54^e^	151.69 ± 5.37^d^
caffeic acid	3.75 ± 0.34^c^	0.58 ± 0.04^ab^	169.91 ± 2.67^d^	277.18 ± 2.67^e^
*p*-coumaric acid	1.57 ± 0.27^b^	0.92 ± 0.13^ab^	227.92 ± 4.65^c^	105.13 ± 2.53^d^
ferulic acid	1.56 ± 0.54^a^	1.41 ± 0.54^a^	269.26 ± 5.48^d^	353.94 ± 4.54^f^
sinapinic acid	8.95 ± 1.73^a^	0.11 ± 0.03^a^	136.32 ± 3.76^b^	169.12 ± 1.65^b^
rosmarinic acid	3.44 ± 1.35^a^	4.14 ± 0.97^a^	778.65 ± 7.65^e^	199.47 ± 4.54^c^
chlorogenic acid	2.26 ± 0.87^c^	ND	271.83 ± 5.12^e^	ND
rutin	7.96 ± 2.75^c^	0.22 ± 0.05^a^	219.65 ± 5.67^e^	461.64 ± 4.89^f^
myricetin	3.41 ± 0.75^b^	1.01 ± 0.43^a^	335.11 ± 3.76^f^	143.66 ± 2.52^e^
naringenin	1.59 ± 0.32^a^	1.12 ± 0.17^b^	737.06 ± 7.23^f^	122.46 ± 2.85^d^
*Morus nigra* L	mg/g	mg/g	μg/g	μg/g
gallic acid	1.60 ± 0.07^a^	2.05 ± 0.10^a^	87.57 ± 3.46^c^	260.47 ± 4.32^f^
caffeic acid	1.20 ± 0.12^b^	0.51 ± 0.01^a^	471.19 ± 0.63^ g^	336.17 ± 1.98^f^
*p*-coumaric acid	0.17 ± 0.02^a^	0.73 ± 0.02^a^	228.91 ± 4.65^c^	184.12 ± 2.34^e^
ferulic acid	1.43 ± 0.09^a^	1.79 ± 0.54^b^	167.81 ± 5.86^c^	287.39 ± 3.57^e^
sinapinic acid	0.99 ± 0.01^a^	1.32 ± 0.56^a^	797.49 ± 5.76^d^	110.47 ± 3.56^c^
rosmarinic acid	0.39 ± 0.03^a^	2.53 ± 0.93^a^	250.42 ± 5.78^d^	111.44 ± 1.97^b^
chlorogenic acid	0.21 ± 0.04^a^	1.73 ± 0.36^b^	48.82 ± 1.74^d^	291.78 ± 4.75^f^
rutin	0.38 ± 0.01^a^	1.55 ± 0.43^b^	522.06 ± 5.11^ g^	115.21 ± 1.41^d^
myricetin	0.89 ± 0.02^a^	1.09 ± 0.43^a^	51.31 ± 3.87^c^	65.55 ± 2.59^d^
naringenin	1.44 ± 0.11^a^	1.51 ± 0.14^a^	139.24 ± 2.64^e^	109.61 ± 2.97^c^

Arithmetic means followed by the same letter within a row indicate no significant difference (*p* < 0.05) in Tukey test

*ND* not detected

Zou et al. [30] found that hydroethanolic extracts of Chines mulberry leaves have the higher concentrations of RUT (from 0.4 to 1.2 mg/g DW) than those found in this study. Kim et al. [27] determined 3.20 mg RUT/g DW in methanolic extracts of Korean white mulberry, while Kutsabe et al. [31] determined 573 mg RUT/100 g DW in hydroethanolic extracts of Japanese white mulberry leaves. In hydromethanolic extracts of white mulberry leaves the CGA was not determined, while He et al. [32] found this phenolic acid on the level from 4.10 to 7.70 mg/g DW in hydromethanolic extracts of Chinese white mulberry leaves. These differences can be assigned to different climatic and environmental conditions (temperature, altitude, soil, humidity, UV) at which the plant grows [33].

### Antioxidant activity

The antioxidant activity is an important parameter for establishing the health benefits of food products [30] and can be quantified by different methods. Among these methods DPPH and FRAP tests are recommended as rapid, simple, low-cost and reproducible tools for measuring the antioxidant potential of plant extracts [34]. The results of DPPH and FRAP assays of the antioxidant activity of mulberry leaves were compiled in Table 2 (Fig. 1S). Generally, white mulberry samples were characterized by the higher antioxidant properties compared to black mulberry samples. For example, FRAP values of aqueous extracts of white mulberry were several times higher than those of black mulberry. Moreover, no significant differences were found (*p* < 0.05) between antioxidant activity of white and black mulberry alcoholic extracts.

Literature data showed that Polumackanycz et al. [20] and Memon et al. [28] reported lower values of DPPH for white mulberry leaves, 52.41–98.82 mg TE/100 g DW and 65.99 μmol quercetin equivalent/100 g, respectively. However, Sanchez-Salcedo et al. [7] determined higher values of DPPH for water extracts of white and black mulberry, from 11.17 to 12.64 and from 10.62 to 12.15 mg TE/g, respectively. The results of the FRAP assays for water extracts of white mulberry leaves are similar to those obtained previously [20] (5.96–21.21 mmol Fe^2+^/g DW), while for hydroalcoholic they are lower [20] (18.10–37.85 mmol Fe^2+^/g DW).

### Correlation Analysis

To elucidate the relationship between the phenolic composition and antioxidant activity of white and black mulberry extracts, Pearson’s correlation coefficient analysis was used (Table 1S). The results showed that the antioxidant properties of white and black mulberry are strongly correlated to TPC, TFC and TPAC (r > 0.92), and to TPC, TFC and ASA (r > 0.94), respectively. It could be concluded that TPC, TFC, TPAC and ASA greatly contribute to the DPPH and FRAP values of mulberry extracts. Several studies also reported a good correlation between antioxidant potential and both TPC and TFC for other plants [35]. The relationship between the individual phenolic compounds and the antioxidant potential revealed that FA and RA in white mulberry extracts and GA, FA and MYR in black mulberry extracts strongly correlated with the FRAP values (r > 0.90). This suggests the crucial role of phenolic compounds as antioxidant constituents in the mulberry extracts. Moreover, no significant correlations were found between DPPH and FRAP values for all analyzed extracts. This might be due to the antioxidant potential against free radicals and inevitably does not equal with its ability to reduce ferric to ferrous.

The correlation coefficients between the levels of individual phenolic compounds in mulberry extracts under study showed that their values are statistically significant (*p* < 0.05) for 14 and 11 pairs of constituents in white and black mulberry extracts, respectively. The highest correlation coefficients were found in the pairs GA-*p*CA (r = 0.98), CA-RUT (r = 0.97), SA-CGA (r = 0.97), CGA-RUT (r = 0.96) in white mulberry extracts, and in the pairs GA-FA (r = 0.96), GA-MYR (r = 0.97) and MYR-NAR (r = 0.96) in black mulberry extracts. Besides, in white mulberry extracts FA and RA were strongly correlated to TPAC (r > 0.92). In black mulberry extracts GA, FA, MYR and NAR were correlated with TPC and TFC (*p* < 0.05; r > 0.90), while RA was correlated with ASA (*p* < 0.05; r > 0.93). Moreover, a strong (r > 0.96) correlation between TPC and TFC was observed in all analyzed mulberry extracts.

## Conclusions

White and black mulberries are plants described as a food with several health benefits. Their bioactivity has been mostly related to their fruits, while studies with leaves are scarce. In this study, leaves of two mulberry species (*Morus alba* L. and *Morus nigra* L.) were characterized, compared and evaluated in terms of their phenolic composition and antioxidant activity. Data obtained showed that, in general, black mulberry leaves were richer in TFC and TPAC, while water extracts of white mulberry leaves contained higher levels of the most individual phenolic compounds. The measurements revealed that water extracts obtained from leaves of both species of mulberry were richer in phenolic constituents and were characterized by higher values of TPC, TFC, TPAC, and L( +)-ascorbic acid content than their alcoholic extracts. Among the individual phenolic constituents, GA was found in the highest concentrations in all leave extracts, while CGA was not detected in alcoholic extracts of white mulberry. Moreover, correlation analysis showed significant relationships between antioxidant activities and both TPC and TFC in white and black mulberry extracts. This fact suggests the crucial role of phenolic compounds as antioxidant agents in white and black mulberry leaves. Data obtained in this study demonstrated that the consumption of a mulberry leaves in the form of teas or other beverages can be beneficial to human health. Moreover, owing to their particular phenolic composition, they can be considered as promising sources of phytochemical compounds with proven biological activities. Leaves can also be used in pharmaceutical and food industry as a source of new and safer bioactive compounds.

## Supplementary Information

Below is the link to the electronic supplementary material.

Supplementary file1 (DOC 477 KB)

## Data Availability

All data generated or analysed during this study are included in this published article.

## Abbreviations

FRAP: Ferric reducing power
DPPH: Free radical scavenging capacity
DW: Dry weight
GAE: Gallic acid equivalent
TE: Trolox equivalent
CAE: Caffeic acid equivalent
ASA: Ascorbic acid
TPC: Total phenolics content
TFC: Total flavonoids content
TPAC: Total phenolic acids content
