# Supplementary material for: Morus alba L. and Morus nigra L. Leaves as a Promising Food Source of Phenolic Compounds with Antioxidant Activity
Source: Plant Foods Hum Nutr. 2021 Sep 27;76(4):458–65. doi: 10.1007/s11130-021-00922-7 (PMC8629867; doi:10.1007/s11130-021-00922-7)
Supplement: Supplementary file 1 — Supplementary file1 (DOC 477 KB) [file 11130_2021_922_MOESM1_ESM.doc]

**SUPPLEMENTARY MATERIAL**

**Article Title**: *Morus alba* L. and *Morus nigra* L. leaves as a promising food source of phenolic compounds with antioxidant activity

**Journal name**: Plant Foods for Human Nutrition

**Author names**: Milena Polumackanycz, Marek Wesolowski, Agnieszka Viapiana*

**Affiliation and e-mail address of the corresponding author**: Department of Analytical Chemistry, Medical University of Gdansk, Gen. J. Hallera 107, 80-416 Gdansk, Poland

E-mail address: [agnieszka.viapiana@gumed.edu.pl](mailto:agnieszka.viapiana@gumed.edu.pl)

**Determination of phenolics and L(+)-ascorbic acid content**

Total phenolic content (TPC) was determined using the Folin-Ciocalteu method described by Singleton and Rossi [1] with some modification. An appropriate amount of sample solution was mixed with 0.2 mL of Folin-Ciocalteu reagent, and 2 mL of 7% Na_2_CO_3_ (w/v) was added after 3 min. After 1 h of incubation at room temperature, absorbance was read at 760 nm. The calibration curve of gallic acid (linearity range: 25-85 mg/mL, r = 0.995) was used to express the results in mg gallic acid equivalent per g dried weight of sample (mg GAE/g DW).

Total flavonoid content (TFC) was determined according to the method described in the European Pharmacopoeia [2] with some modifications. Briefly, an appropriate amount of the sample solution was mixed with 0.1 mL of 5% aluminium chloride and 1.4 mL of a mixture of acetic acid and methanol (1:19, v/v). After incubation at room temperature for 30 min, absorbance was measured at 425 nm. The TFC was expressed as µg of quercetin equivalent per g dried weight of sample for (µg QE/g DW) using a calibration curve of the quercetin standard solutions (linearity range: 20-50 µg/mL, r = 0.997).

Total phenolic acid content (TPAC) was determined using Arnov’s reagent according to the procedure described in the Polish Pharmacopoeia VI [3]. An appropriate amount of sample solution was mixed with 0.2 mL 0.5 M HCl, 0.2 mL of Arnov’s reagent (10 g sodium molybdate and 10 g sodium nitrite in 100 mL of redistilled water) and 0.2 mL 1 M NaOH. Absorbance was measured at 490 nm. The results were expressed as µg of caffeic acid equivalent per g dried weight of sample (µg CAE/g DW) based on a calibration curve of caffeic acid (linearity range: 4.8-33.6 µg/mL, r = 0.995).

L(+)-ascorbic acid content (ASA) was determined using the Abdelmageed method [4]. An appropriate amount of sample solution was mixed with 0.2 mL 0.2 M of NaOH, 0.2 mL of 4-chloro-7-nitrobenzofurazane (NBD-Cl) and 1.4 mL of 50% (v/v) aqueous acetone solution. After 30 min, absorbance was read at 582 nm against a blank (50% aqueous acetone solution, v/v). Using an ascorbic acid standard curve (linearity range: 8.0-35.2 µg/mL, r = 0.998), the content of L(+)-ascorbic acid of the extract was expressed as mg of ascorbic acid per g dried weight of sample (mg ASA/g DW).

**DPPH scavenging activity assay and FRAP assay**

The DPPH radical scavenging activity was assessed according to a modified method of Tuberoso et al. [5]. Briefly, an appropriate amount of sample solution was added to 2.8 mL of methanolic DPPH solution (100 µmol/L) and after 30 min the absorbance was measured at 517 nm. Quantitation was performed using the external standard method at a linearity range of 100-320 mg/mL. The data obtained were expressed in mg Trolox equivalent per g dried weight of sample (mg TE/g DW).

Ferric reducing/antioxidant power (FRAP) assay was performed using the method proposed by Benzie and Strain [6]. An appropriate amount of sample solution was mixed with 2.3 mL of FRAP reagent (0.2 mol acetate buffer: 20 mmol FeCl_3_ : 10 mmol TPTZ; 10:1:1; v/v/v). After incubation at room temperature for 30 min, absorbance was measured at 593 nm. A calibration curve at a range of 85-600 µmol/L of ferrous sulphate was employed and results expressed in mmol ferrus ion equivalents (Fe^2+^) per g dried weight of sample (mmol Fe^2+^/g DW).

**References**

1. Singleton VL, Orthofer R, Lamuela RRM (1999). Analysis of total phenols and other oxidation substrates and antioxidants by means of Folin-Ciocalteu reagent. Methods Enzymol 299:152-178

2. European Pharmacopoeia. Birkenblätter–Betulae Herba 4.00; Council of Europe: Strasbourg, France, 2002; p. 1308.

3. Polish Pharmacopoeia VI; Polish Pharmaceutical Society: Warszawa, Poland, 2002; p. 150

4. Abdelmageed OH, Khashaba PY, Askal HF, Saleh GA, Refaat IH (1995). Selective spectrophotometric determination of ascorbic acid in drugs and foods. Talanta 42:573-579

5. Tuberoso CIG, Rosa A, Bifulco E, Melis MP, Atzeri A, Pirisi FM, Dessi MA (2010). Chemical composition and antioxidant activities of *Myrtus communis* L. berries extracts. Food Chem 12:1242-1251

6. Benzie IFF, Strain JJ (1996). The ferric reducing ability of plasma (FRAP) as a measure of “antioxidant power”: The FRAP assay. Anal Biochem 239:70-76.


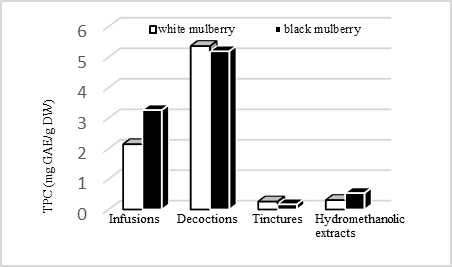

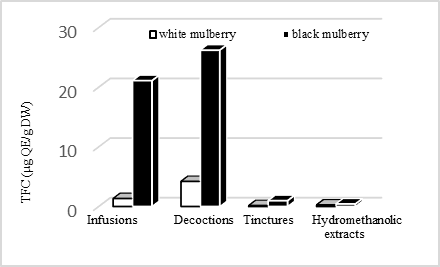


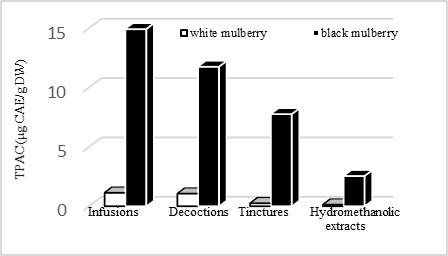

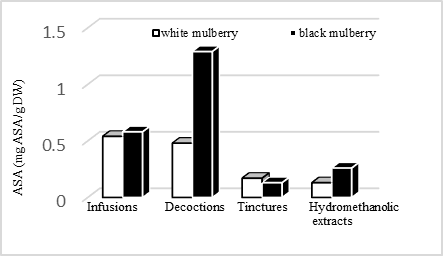


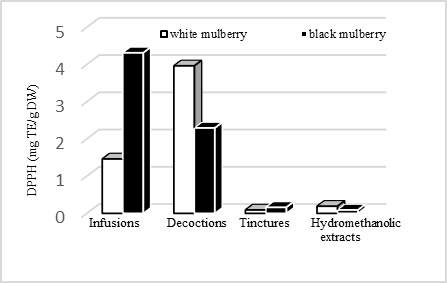

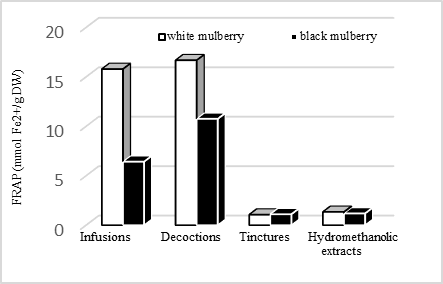


**Figure 1S** Total phenolics content (TPC), total flavonoids content (TFC), total phenolic acids content (TPAC), L(+)-ascorbic content (ASA) and antioxidant activity of white and black mulberry leaves extracts.


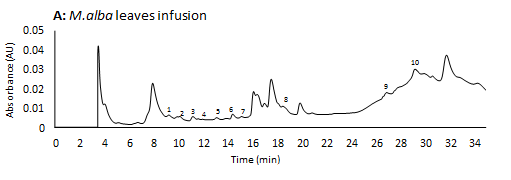


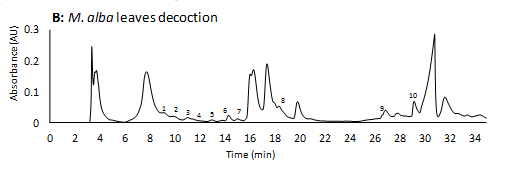


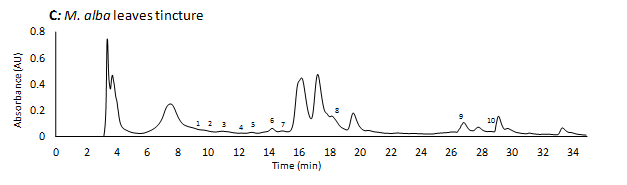


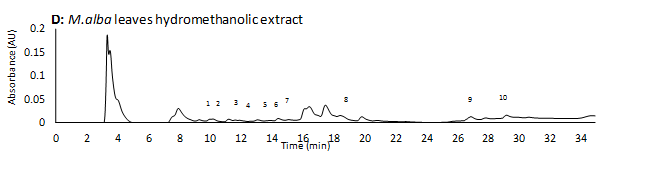


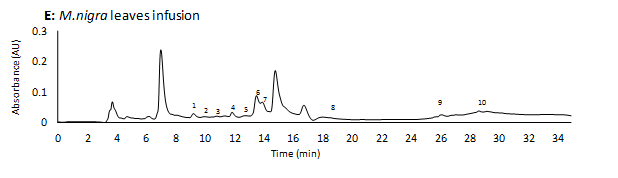


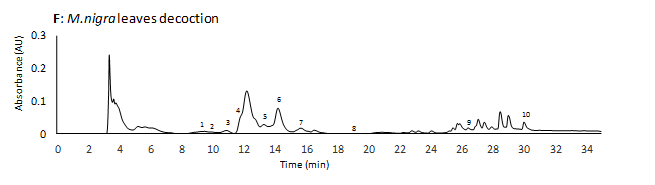


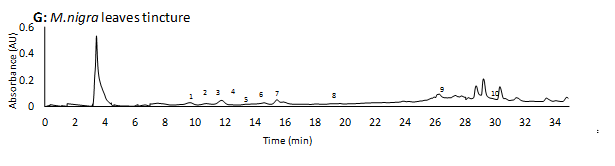


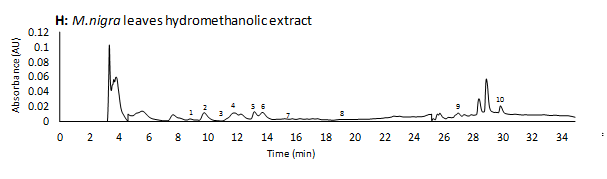


**Figure 2S.** HPLC profiles of the *M. alba* and *M. nigra* leaves extracts recorded with UV-Vis detector: at 280 nm for GA (no 1; 8.71 min), SYN (no 6; 14.22 min), RA (no 8; 18.88 min), NAR (no 9; 26.45 min); at 320 nm for CGA (no 2; 9.71 min), CA (no 3; 10.80 min), *p*CA (no 4; 11.92 min), FA (no 5; 12.86 min); at 370 nm for RUT (no 7; 15.26 min), MYR (no 10; 29.76 min).

**Table 1S** Correlation matrix (only statistical significant correlations) of the phenolic composition and antioxidant activity in mulberry samples.

|  | *Morus alba* L. | | *Morus nigra* L. |
| --- | --- | --- | --- |
|  | DPPH | FRAP | FRAP |
| TPC | 0.95 |  | 0.96 |
| TFC | 0.97 |  | 0.95 |
| TPAC |  | 0.98 |  |
| ASA |  |  | 0.95 |
| GA |  |  | 0.94 |
| FA |  | 0.94 | 0.93 |
| RA |  | 0.95 |  |
| MYR |  |  | 0.91 |

*p* < 0.05
